# Promiscuous Gene Expression in the Thymus: A Matter of Epigenetics, miRNA, and More?

**DOI:** 10.3389/fimmu.2015.00093

**Published:** 2015-03-03

**Authors:** Olga Ucar, Kristin Rattay

**Affiliations:** ^1^Division of Developmental Immunology, German Cancer Research Center, Heidelberg, Germany

**Keywords:** mTEC, promiscuous gene expression, Aire, epigenetic, miRNA, tolerance, tissue-restricted antigen

## Abstract

The induction of central tolerance in the course of T cell development crucially depends on promiscuous gene expression (pGE) in medullary thymic epithelial cells (mTECs). mTECs express a genome-wide variety of tissue-restricted antigens (TRAs), preventing the escape of autoreactive T cells to the periphery, and the development of severe autoimmunity. Most of our knowledge of how pGE is controlled comes from studies on the autoimmune regulator (Aire). Aire activates the expression of a large subset of TRAs by interacting with the general transcriptional machinery and promoting transcript elongation. However, further factors regulating Aire-independent TRAs must be at play. Recent studies demonstrated that pGE in general and the function of Aire in particular are controlled by epigenetic and post-transcriptional mechanisms. This mini-review summarizes current knowledge of the regulation of pGE by miRNA and epigenetic regulatory mechanisms such as DNA methylation, histone modifications, and chromosomal topology.

## Introduction

The establishment of central tolerance to all organs of the body is to a large extent mediated by the unique ability of medullary thymic epithelial cells (mTECs) to express a vast variety of self-antigens. This so-called promiscuous gene expression (pGE) encompasses a genome-wide selection of tissue-restricted antigens (TRAs), and so far no involvement of tissue-specific transcription factors in their regulation has been observed in the thymus (1, 2). pGE sets the scope of self tolerance, i.e., clonal deletion and Treg induction, and faulty thymic expression of even a single TRA can precipitate organ-specific autoimmunity (3–5); however, we still lack a coherent model incorporating and explaining all the intricacies of pGE.

Most of our knowledge of the molecular control of pGE comes from studies on autoimmune regulator (Aire) (6, 7). Mutations in the *AIRE* gene cause a rare monogenic autoimmune disorder autoimmune polyendocrinopathy–candidiasis–ectodermal dystrophy (APECED), affecting multiple organs with a preference for endocrine glands (8, 9). The Aire-deficient mouse model recapitulates the autoimmune phenotype observed in human patients (10).

Autoimmune regulator controls the expression of a subset of TRAs by interacting with the general transcriptional machinery and promoting transcript elongation. Aire does not have a dedicated DNA recognition motif, and it is unclear how it is targeted to an exclusive set of TRA-encoding genes, which is largely conserved across species (11). Depending on the cellular context, Aire can induce the expression of different sets of genes (12), suggesting that the epigenetic landscape of mTECs plays a role in defining Aire targets. Moreover, many TRAs are expressed in TECs in an Aire-independent manner implying that additional factors also regulate pGE. Noteworthy, a set of cell-lineage-specific TFs has proven dispensable for promiscuous transcription of the corresponding target genes in the thymus (13–16). Thus, the likelihood of tissue-specific TFs responsible for the Aire-independent gene regulation in mTECs or TFs acting in concert with Aire remains an open question.

Recent studies documented a role for epigenetic and post-transcriptional mechanisms in regulating pGE in general and the function of Aire in particular (14, 17–19). Indeed, both APECED patients (8) and mouse mutants (20) display variability in the disease severity and the organs affected depending on different genetic backgrounds, indicating that other genetic or epigenetic components define the exact course of the individual disease. Here, we briefly review our current knowledge of how DNA methylation, histone modification, and miRNA may influence pGE and mTEC maintenance.

## Epigenetic Regulation of pGE

Transcription factor-triggered gene expression is cross-regulated by a number of enzymes, which modify the DNA itself (DNA methylation) or the histones (histone post-translational modifications). Recent studies showed that DNA and histone modifications can alter the promoter structure and accessibility to the extent of adding new TF binding sites, thus shaping the level and pattern of gene expression and, consequently, developmental decisions (21). Accumulating evidence suggests that all these modifications might also be involved in regulating pGE.

### DNA methylation

Methylation of cytosines in CpG dinucleotides is essential for the regulation of embryonic development, cell lineage progression, gene expression, and chromatin structure (22) and is implicated in several human diseases (23). The majority of CpGs in the human genome are methylated, whereas CpG islands at the transcription start sites of housekeeping genes are hypomethylated (24). As a rule, DNA methylation inversely correlates with gene expression level: a high degree of DNA methylation at promoter regions prevents the binding of transcription factors to their DNA-binding motives and results in transcriptional silencing (21, 22). DNA methylation pattern reflects the developmental status of cells with respect to lineage commitment/progression. Thus, the gene loci of myeloid-specific TFs and their binding sites are hypermethylated in the cells of the lymphoid lineage (25). Consistently, demethylation of promoter regions facilitates lineage-specific gene expression, e.g., CD8^+^ T cell markers are specifically unmethylated and highly expressed in T cells committed to the CD8 lineage (25). Comparison of the methylation patterns of stem/progenitor cells and lineage-committed cells of various tissues demonstrated that the changes in methylation occur at certain lineage-specific gene promoters rather than in extended chromosomal clusters; moreover, this specific methylation pattern is maintained in cells and defines their identity (25–27).

**Figure 1:**
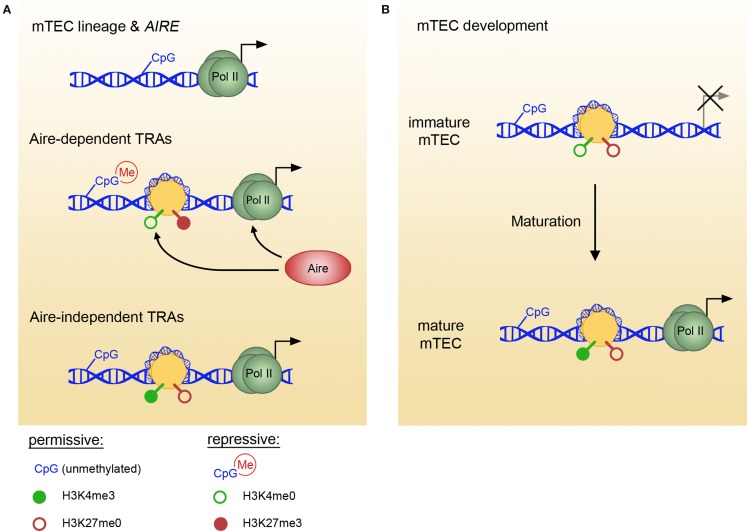
**Epigenetic marks in mTECs vary between TRA pools and with mTEC maturation**. **(A)** In mTECs, the loci of TEC lineage-specific genes and various TRA pools bear different epigenetic marks. Thus, lineage genes and *AIRE* (top) are characterized by low DNA methylation content (permissive), Aire-dependent genes (middle) favor repressive marks, whereas Aire-independent genes (bottom) are maintained in a permissive state. Aire specifically binds H3K4me0, facilitating polII recruitment and transcript elongation. **(B)** Aire-independent TRA loci undergo epigenetic changes with mTEC maturation, indicating that permissive marks are acquired in a stepwise fashion and maintained before the onset of gene expression. For example, in immature mTECs, *Csn2* genomic locus exists in an epigenetically neutral state, and the appearance of permissive H3K4me3 modification coincides with mTEC maturation and the onset of transcription. TRAs, tissue-restricted antigens; mTEC, medullary thymic epithelial cell.

Several recent reports on DNA methylation profiles in mTECs suggest that this epigenetic modification might also pertain to the control of mTEC lineage commitment and pGE; however, the exact specificity and significance of DNA methylation remains unclear (Figure 1A). A number of TEC-specific genes are hypomethylated in mTECs in contrast to other thymic cell types and peripheral tissues (28), suggesting that this pattern arises during lineage commitment and defines mTEC cellular identity. *In vitro* and *ex vivo* studies demonstrated that the *AIRE* gene promoter is hypomethylated in mTECs (29, 30), which indicates its transcription-permissive state. In contrast, the *Fgg* (fibrinogen gamma chain) gene, which encodes a liver-specific protein promiscuously expressed in an Aire-dependent manner, is hypermethylated in both Aire-expressing and Aire-deficient mTECs (28). Further intriguing results were obtained from the comparison of the DNA methylation state of the casein locus and the *Gad1* (Gad67) promoter between immature and mature adult mTECs (14). The DNA methylation pattern of both regions exists in a permissive state already in immature mTECs, presumably allowing rapid promoter activation after mTEC maturation (Figure 1B). Interestingly, the *Csn2* promoter showed progressive demethylation in mature mTECs during embryonic development, which preceded the onset of gene expression (14). Together with the reported hypermethylation of *Fgg* locus (28), these observations suggest that DNA methylation pattern of TRA loci in mTECs does not reflect the promoter activity in the same way as in other peripheral tissues. In summary, although mTECs seem to adapt the “peripheral” DNA methylation pattern for their lineage-specific genes, they might employ a different strategy for genes expressed promiscuously.

### Histone modification

Post-translational modifications of histone N-terminal tails are known to be essential in the regulation of transcription, chromatin structure, DNA repair and replication, and alternative splicing. They control gene expression by both recruiting effector proteins, the so-called readers of histone marks, and through changing the compaction state of the chromatin (21, 31). Histone modifications fall into active (promoting transcription) and repressive marks and are associated with different chromatin compaction states (32). Active histone marks at promoter and enhancer regions influence polII assembly and elongation through interaction with the basic transcriptional machinery: H4 acetylation and H3 trimethylation at K4 are recognized by TFIID and mediate polII assembly at promoters (33, 34).

In some instances, active and repressive marks can co-exist within the same region. Thus, embryonic stem cells display overlapping repressive and permissive histone modifications at developmental genes, maintaining their inactivity in steady state, but allowing for rapid activation when differentiation starts (35). Other examples of a poised, bivalent histone code at lineage-specific gene promoters have been observed in CD4^+^ T cell lineages (36). The coexistence of active (H3K4me3) and repressive (H27K4me3) marks at promoters of lineage-defining transcription factors GATA3, Tbet, Rorc, and Foxp3 endows different CD4^+^ T cell subtypes with the plasticity to rapidly cross-differentiate into another subtype in response to environmental stimuli (37).

Studies of histone modifications in mTECs indicate that the histone code plays a role in the regulation of pGE, although the exact mechanisms employed might differ for different sets of TRAs (Figure 1A). Aire-dependent genes in mTECs are characterized by a lack of H3K4 trimethylation and enrichment in repressive H3K27me3 (17, 38). Aire has been shown to specifically bind to unmethylated H3K4, targeting genes in a state of low H3K4me3 or H3 acetylation (17). Aire binding was correlated with an increase in H3K4me3 and polII recruitment to Aire-dependent gene promoters, implying that Aire facilitates the establishment of active histone marks (17). After polII assembly, Aire assists in the transcriptional elongation through facilitating p-TEFIIb recruitment (39, 40). Since p-TEFIIb recruitment requires active histone marks at the enhancer regions (41), involvement of Aire in reading enhancer histone code remains an intriguing possibility.

In contrast to Aire-dependent genes, Aire-independent TRAs seem to favor permissive histone modifications (38). In a recent study, Kyewski and colleagues assessed the chromatin state in mature and immature mTECs at two specific loci encoding Aire-independent TRAs, namely *Csn2* and *Gad1* (14). Both gene promoters were characterized by permissive histone marks; furthermore, in the case of the *Csn2* promoter, H4 acetylation and H3K4me3 marks increased with mTEC maturation (Figure 1B). Interestingly, repressive H3K27me3 marks were absent from *Csn2* promoter in both immature and mature mTECs, suggesting that at least some TRA promoters maintain a neutral rather than repressed steady state in immature mTECs (14). Whether a similar poising of chromatin occurs in Aire-dependent gene loci early in mTEC lineage progression remains to be determined.

In summary, the histone code of TRA promoters in mTECs can exist in either permissive (Aire-independent genes) or bivalent (Aire-dependent genes) states. One of many functions of Aire seems to be reading and modifying the histone marks, but it is still unclear how their initial deposition is regulated. To this end, functional studies of chromatin modifiers in TECs should shed new light on the mechanisms mTECs employ to achieve and maintain a transcriptionally poised state of TRA-coding genes. It is also pertinent to understand the epigenetic differences between Aire-dependent and -independent genes, which might lead to the identification of factors controlling Aire-independent pGE.

### Epigenetic landscape flexibility in mTECs

Recent studies revealed that DNA methylation and histone modifications function in a cooperative manner to re-shape chromatin (21, 42), and promoter activity can be predicted by the combination of both (43). The stability of transcriptionally active sites largely depends on the cellular and developmental context; more changes in the epigenetic landscape occur during development and lineage progression than in terminally differentiated cells (41). Is this also the case for pGE? On the population level, mTECs express thousands of genes promiscuously, but their global DNA methylation profile is not significantly different from that of peripheral tissues (28). One should, however, consider that individual TRAs are expressed by only a minor fraction of mTECs [1–3% on average; (38, 44)]. This might result in an under-representation of TRA-specific epigenetic marks in a population analysis. In this respect, studies of single TRA loci within the mTEC subpopulations expressing that specific TRA will be more informative than global epigenomic approaches. Given that pGE increases in its complexity during mTEC maturation and that even in mature mTECs the expression of individual TRAs seems to be transient (45), the epigenetic landscape in the thymus might turn out to be more flexible and dynamic than in other tissues.

Thus, in the case of the casein locus DNA methylation pattern is established before the histone code and both exist in a permissive state even before Csn2 mRNA can be detected [Ref. (14); Figure 1B]. Since Csn2 seems to be somewhat special with regard to its expression frequency in mTECs (44), the sequential establishment and cooperation of CpG context and histone marks should be examined in other, less frequent TRA loci at different stages of mTEC development. The interplay between histone marks and DNA methylation pattern can result in the organization of actively transcribed loci in transcriptional factories (46), demonstrating the influence of epigenetic marks on genome topology. Recently, emerging evidence suggests that higher order interactions between chromosomal regions in cis and trans might impose further influence on gene expression (47, 48). Importantly, stochastic interchromosomal interactions can account for gene expression heterogeneity in a population: for example, co-localization of β-globin gene and its enhancer was observed in 5–10% of cells in a population and correlated with a ~100-fold increase of β-globin expression in these “jackpot” cells (49). The fact that only a small proportion of mTECs expresses a given TRA at a given time together with a recent observation of TRA loci co-localization (45) suggests that such higher order organization might regulate patterns of gene co-expression in single mTECs in the context of pGE.

## Post-Transcriptional Regulation of pGE

**Figure 2:**
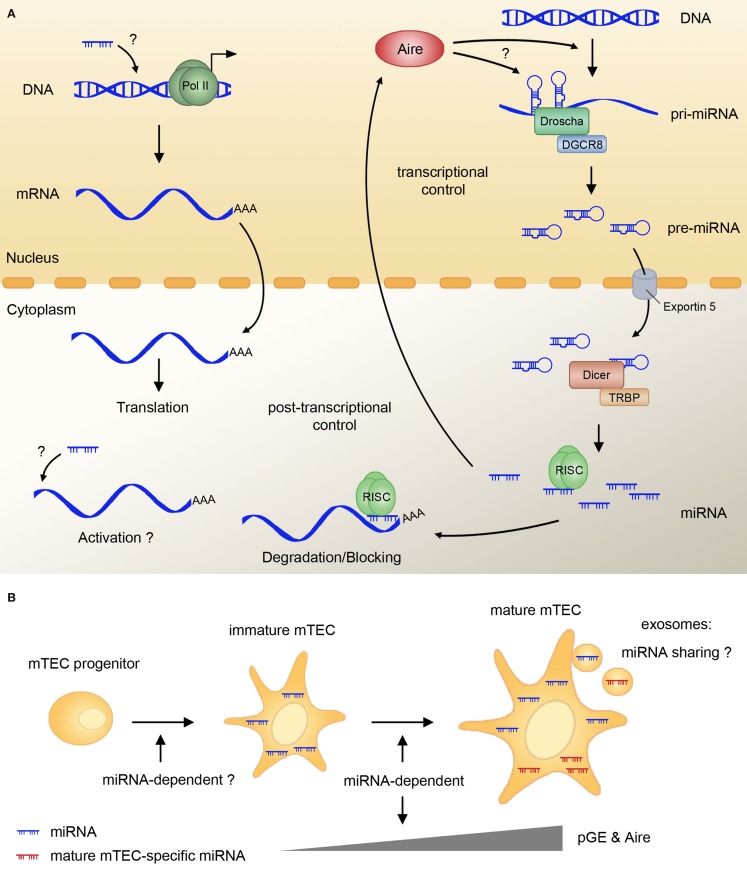
**miRNAs regulate pGE and mTEC maturation**. **(A)** The majority of miRNA primary transcripts (pri-miRNA) are generated by polII and further processed by Drosha/DGCR8 in the nucleus to the stage of pre-miRNA. Pre-miRNA are exported into the cytoplasm by Exportin 5, and further cleaved by the Dicer complex, resulting in mature miRNA. These can be incorporated into the RNA-induced silencing complex (RISC), which binds the target mRNA and usually mediates translational block and/or mRNA degradation. In mTECs, miRNA are indispensable for the expression of Aire and Aire-dependent and -independent TRAs. The mechanism of miRNA action in pGE is not known, and it might involve activation of transcription or translation of TRAs. Aire might be involved in regulating the expression of miRNA-encoding genes and in the generation of miRNA precursors from the so-called miRtrons. **(B)** mTEC maturation and pGE rely on an intact miRNA pathway, as Dicer deletion in TECs blocks different stages of mTEC lineage progression. Several miRNA are specifically upregulated upon mTEC maturation; however, their exact function and the influence they exert on TRA and Aire expression remains to be determined. The fact that mTEC-specific miRNA are found in human thymic exosomes suggest the possibility of mTECs sharing these small regulators with other antigen-presenting cells in the course of central tolerance induction. RISC, RNA-induced silencing complex; mTEC, medullary thymic epithelial cell; pGE, promiscuous gene expression.

miRNA represents a class of small (≈22 nt) RNA molecules involved in the post-transcriptional control of gene expression, acting as switches and fine-tuners of translation (50). Primary miRNA transcripts are polII-dependent and undergo two steps of post-transcriptional processing: by Drosha and DGCR8 in the nucleus and by Dicer and TRBP in the cytoplasm [Ref. (51); Figure 2A]. The mature miRNA are incorporated into the RNA-induced silencing complex (RISC), binding of which to the target mRNA causes a translational block and subsequent mRNA decay (52, 53). Target recognition depends on a sequence-specific interaction between the target mRNA 3′UTR and the seed sequence of miRNA (54). More than half of all protein-coding genes in mammals are regulated by miRNA, and many of them have binding sites for several unrelated miRNA in their 3′UTR. Additional complexity arises from the fact that a single miRNA can affect the expression of multiple targets. Studies of miRNA function in various tissues revealed complex balanced miRNA–mRNA interaction networks, regulating tissue homeostasis, cell fate decisions, and disease progression (55).

Several recent studies suggest that miRNA may be involved in the regulation of pGE. We showed that a number of miRNA exhibit subset-specific expression in TECs isolated from murine or human thymus; a substantial overlap between miRNA signatures of both species suggests that miRNA expression profiles in TECs are evolutionarily conserved (18). We also demonstrated that maturation-dependent expression of certain miRNA in mTECs correlates with Aire expression (18). Furthermore, changes in miRNA signature have been reported upon Aire knockdown in cell culture (56) and in Aire null mutant thymi (18). Whether Aire directly regulates transcription of miRNA-encoding genes in mTECs and whether such a regulation is a part of stochastic pGE remains to be determined. Since miRNA-encoding genes can use alternative promoters and many miRNA are located in introns (57, 58), Aire might be involved in the direct control of miRNA transcription as well as in the miRNA biogenesis coupled to host mRNA processing. Interestingly, Aire has been implicated in Lin-28-dependent regulation of let-7 miRNA in ES cells (59). As for Aire being regulated by miRNA, a recent report showed that Aire expression could be controlled by miRNA-220b in an artificial cell culture system (60). It is unclear whether this regulation occurs in human or mouse mTECs *in vivo*, and no conserved miRNA target sites in Aire mRNA have been predicted *in silico* by the currently available target prediction tools.

miRNA expression in the thymic epithelium is indispensable for the establishment of central tolerance. Thus, TEC-specific ablation of Dicer or DGCR8 (and therefore all mature miRNA) leads to premature thymic involution, diminished T cell output and increased susceptibility to autoimmune disease (18, 61–63). The lack of Dicer in TECs leads to a dramatic decline in pGE – a possible underlying cause of the breach in central tolerance. Interestingly, pGE decline affects both Aire-dependent and -independent TRAs in mTECs and cTECs (18), and precedes the loss of TEC cellularity (18, 62). The premature involution phenotype of Dicer and DGCR8 mutants is recapitulated in the mouse model lacking miR-29a (61). However, these latter mutants do not exhibit the defects in epithelial organization that result from the loss of all canonical miRNA (61, 63) and show only mild delayed impairment of Aire-dependent pGE (18), suggesting that miRNA other than miR-29a play a role in TEC maintenance and function. Further investigation of the mTEC-specific miRNA and their targets will be needed to comprehend the miRNA-dependent regulation of pGE.

How do post-transcriptional inhibitors facilitate pGE? First, in rare cases, miRNA were shown to activate rather than repress gene expression, e.g., through binding to the 5′-UTR [Ref. (64); Figure 2A]. Gene expression activation can also happen indirectly after the miRNA-mediated downregulation of proteins involved in transcriptional repression or RNA decay (53, 65). Finally, miRNA could affect pGE indirectly by promoting the maturation of mTECs. Indeed, FoxN1–Cre-mediated loss of Dicer causes alterations in mature mTEC surface antigen profiles (18) and might impair the early stages of mTEC lineage progression (Figure 2B). The fact that mTEC lineage progression and terminal differentiation seem to be unaffected in miR-29a mutants (18) suggests that other miRNA play a role in these processes. Of note, stemness, differentiation, and senescence of keratinocytes seem to be controlled by a complex network of p63 and several miRNA (66). Given the close parallels between keratinocyte and mTEC differentiation (67) further studies on miRNA function in the thymus should reveal whether a similar network determines turnover, maintenance, and function of mTECs.

Apart from being required for mTEC development and pGE, TEC-specific miRNA might play a role in other mechanisms of central tolerance establishment. One of these mechanisms involves a transfer of TRAs from mTECs to dendritic cells (68). Though it is unclear by which precise means the antigens are shared, exosome transfer is a possible route (69). Intriguingly, a recent study showed that human thymic exosomes contain TRAs and TEC-specific miRNA (70). miRNA transfer via exosomes was shown to be functionally relevant in various settings. Thus, T cells share their miRNA by this pathway with antigen-presenting cells and other T cells (71, 72). Whether transfer of miRNA from mTECs to dendritic cells indeed takes place via exosomes and the functional significance of this exchange will be clarified in future studies.

## Concluding Remarks

Promiscuous expression of peripheral antigens in the thymus keeps autoimmunity at bay; grasping its exact molecular mechanism will lead to a better understanding of how central tolerance is established and maintained. Transcription factor-mediated gene expression has been shown to go hand in hand with epigenetic and post-transcriptional regulation in many peripheral tissues. Recent studies of these modes of regulation in mTECs suggest that epigenetic marks are deposited and interpreted in an unconventional way in the course of pGE, and that miRNA play an important role in maintaining TRA expression. The future challenge lies in finding out how exactly mTECs utilize ubiquitous epigenetic and post-transcriptional mechanisms to achieve and maintain their extraordinarily broad expression profiles. Will pGE eventually turn out to employ a unique scenario of gene regulatory modes for the sake of preserving tolerance?

## Conflict of Interest Statement

The authors declare that the research was conducted in the absence of any commercial or financial relationships that could be construed as a potential conflict of interest.
